# Crosstalk between MicroRNA and Oxidative Stress in Physiology and Pathology

**DOI:** 10.3390/ijms21041270

**Published:** 2020-02-13

**Authors:** Antonella Fioravanti, Luigi Pirtoli, Antonio Giordano, Francesco Dotta

**Affiliations:** 1 Department of Medicine, Surgery and Neuroscience, Rheumatology Unit, Azienda Ospedaliera Universitaria senese, Policlinico Le Scotte, 53100 Siena, Italy; 2 Sbarro Institute for Cancer Research and Molecular Medicine, Department of Biology, College of Science and Technology, Temple University, Philadelphia, PA 19122, USA; luigipirtoli@gmail.com (L.P.);; 3 Department of Medical Biotechnologies, University of Siena, 53100 Siena, Italy; 4 Diabetes Unit, Department of Medicine, Surgery and Neurosciences, University of Siena, Policlinico Le Scotte, 53100 Siena, Italy; francesco.dotta@unisi.it

MicroRNAs (miRNA), are short regulatory RNA molecules that regulate gene expression by binding specific sequences within target messenger RNA (mRNA). Increasing evidence revealed their involvement in important physiological cellular processes as well as in the pathophysiology of different disorders, including cancer, cardiovascular diseases, diabetes mellitus, and rheumatic and neurological disorders.

miRNA in different body fluids are considered new candidate biomarkers for diagnosis, classification, prognosis, and responsiveness to treatment, although none have been proposed for daily clinical use. Furthermore, the development of therapeutic strategies either restoring or repressing miRNA expression and activity has attracted much attention. Notwithstanding miRNA have been extensively studied, their detailed mechanisms of action have not yet been fully understood.

Increasing evidence has shown a crosstalk between miRNA and components of redox signaling. miRNA may regulate the expression of redox sensors and other reactive oxygen species (ROS) modulators, such as the key components of cellular antioxidant machinery, while ROS can induce or suppress miRNA expression and contribute to downstream biological function through the regulation of target genes.

The Special Issue entitled “Crosstalk between MicroRNA and Oxidative Stress in Physiology and Pathology” of the International Journal of Molecular Sciences includes three Original Articles and eleven Reviews providing new insights on the interaction between miRNA and oxidative stress under normal and diseased conditions.

A Review by Tsai et al. [1] discusses the crosstalk between excessive oxidative stress induced by mitochondrial dysfunction in tissues/cells and noncoding RNAs, highlighting the role of the epigenetic modulation and of the antioxidant therapy as possible new therapeutic strategies for patients with systemic lupus erythematosus.

Cheleschi et al. [2], in an in vitro study on human osteoarthritic synovial fibroblasts, confirm the presence of a complex relationship between the adipokines, visfatin, resistin, and some miRNA (miR-34a, miR-146a, and miR-181a) in the regulation of oxidative stress balance.

Furthermore, a study on rat cardiomyoblast cells performed by Zhang et al. [3] identifies miR-27a-5p as a cardioprotective agent on hypoxia-induced H9c2 cell injury, suggesting it may be a novel target for the treatment of hypoxia-related heart diseases.

Klieser et al. [4] provide a comprehensive overview of the interactions of oxidative stress and miRNA in pathological processes of the liver. Both, miRNA and oxidative stress are involved in the multifactorial development and progression of acute and chronic liver diseases, and carcinogenesis, by influencing numerous signaling and metabolic pathways.

Quadir et al. [5] extensively review the recent progress in the field of oxidative stress in diabetes mellitus, specifically focusing on the relationship between miRNA and oxidative stress during disease progression as well as on the role of miRNA as candidate biomarkers for the prediction and staging of diabetic chronic complications.

The role of individual miRNA in oxidative stress and related pathways has been further reviewed and confirmed in different neurodegenerative conditions by Konovalova et al. [6], who also raise some criticisms associated with the use of oversimplified cellular models and highlight the ways of studying miRNA regulation and oxidative stress in human stem cell-derived neurons.

A large contribution has been provided on cancer research. Cosentino et al. [7], dealing with Breast Cancer; Huang et al. [8] with Human Hepatocellular Aarcinoma; Zhang [9] for therapeutic tolerance and resistance as a general subject; and Lin. [10] and Babu and Tay [11] on the overall ROS–miRNA relationship domain. All these Authors thoroughly address and analyze from different perspectives the genomic, epigenetic, transcriptional, signaling, and metabolic levels at which the interplay occurs, on the grounds of a systematic and updated check of the evidence emerging from the related literature. Furthermore, Yamakawa et al. [12] address the subject of the possible development of Clinical Trials of Nucleic Acid Medicine, and their delivery systems for Pancreatic Cancer. Of note, among the above quoted contributors, Zhang [9] points out the opportunity of Large-Scale Screenings and Artificial Intelligence-based technology to optimize the therapeutic approach, that is, in accordance with our considerations about complexity in the conclusive remarks. Additionally, a very interesting overview comes from the paper by Marí-Alexandre et al. [13], who analyze the role of oxidative stress and miRNA in the pathophysiology of endometriosis and its possible evolution towards Ovarian Cancers: with their paper, they also provide a valuable educational contribution to this subject. The only oncological original research paper in this Special Issue [14] is dedicated to the overexpressed miR526b/miR655 upregulation of Thioredoxin Reductase 1 (TXNRD1) in Breast Cancer cells, identifying, through a bioinformatic analysis on external datasets, some negative regulators of TXNRD1 as direct targets. Their experiments show that oxidative stress induces miR526b/miR655 overexpression, thus establishing the dynamic function of these miRNA in oxidative stress induction in breast cancer. The adopted in silico procedure has allowed to deepen the knowledge of the involved transcription factors.

It is noteworthy that an exceeding majority of the articles included in this Special Issue—after an extensive call for papers and a rigorous peer-review process—are Reviews of the literature, which are available thanks to the previous, intensive work carried out over many years on miRNA, oxidative stress, and their reciprocal crosstalk. A possible interpretation of this remark is that, at the present time, the involved researchers and scholars are still pondering the overall and ultimate contribution of this scientific domain to the medical sciences. It seems that this field of biology and pathophysiology still preserves an apparent “opacity” regarding its possible practical development, that is, reliable markers and actionable targets for developing a cure. The hallmarks of complexity are as follows: the emergence of unsatisfactorily explained phenomena; the incomplete adequacy of the reductionistic experimental approach; the non-linearity of relationships; dynamic interactive variations. Indeed, complex systems are not completely reducible to direct cause–effect deterministic approaches, and new investigation toolsets are necessary.

The Editors hope that these articles will help readers to update their knowledge about the role of miRNA and oxidative stress in physiology and pathology. Finally, the Editors deeply appreciate all the Authors who contributed excellent Articles to this Special Issue.
